# Anti-CD19 Chimeric Antigen Receptor T Cell Therapy With Tisagenlecleucel for Secondary Central Nervous System Lymphoma: A Case Series

**DOI:** 10.7759/cureus.45088

**Published:** 2023-09-12

**Authors:** Armaan Dhaliwal, Shivtaj Mann

**Affiliations:** 1 Internal Medicine, University of Arizona College of Medicine, Tucson, USA; 2 Hematology and Medical Oncology, University of Arizona Cancer Center, Tucson, USA

**Keywords:** double hit lymphoma, relapsed/refractory, tisagenlecleucel, chimeric antigen receptor t cell therapy, secondary cns lymphoma

## Abstract

Relapsed or refractory (R/R) large B cell lymphoma (LBCL) presenting as secondary central nervous system lymphoma (SCNSL) carries a poor prognosis, with a median survival time of two to five months. Chimeric antigen receptor (CAR)-T cell therapy has been approved in R/R LBCL, but studies are ongoing to understand its efficacy and safety for SCNSL. Axicabtagene ciloleucel or tisagenlecleucel have been shown to attain high response rates in some retrospective studies; however, response durability continues to be unclear. Our study is a case series of three patients with R/R SCNSL who were treated with tisagenlecleucel. One patient achieved a complete response 30 days after CAR-T therapy but developed disease progression on day +100 imaging. The second patient had a partial response and eventual disease progression with ultimately death. The third patient died from central nervous system complications of CAR-T therapy. Two of the three patients developed immune effector cell-associated neurotoxicity syndrome grade 4 and cytokine release syndrome grade 1 toxicities. Our series of three patients demonstrates that R/R SCNSL can elicit a response with CAR-T therapy, although with a limited duration response.

## Introduction

Relapsed or refractory (R/R) large B cell lymphoma (LBCL) with secondary central nervous system lymphoma (SCNSL) carries a dismal prognosis, with a reported median survival of two to five months. High-dose methotrexate (HD-MTX), whole brain radiation therapy (WBRT), and/or high-dose chemotherapy followed by autologous stem cell transplant has been the centerpiece of treatment for these patients in the past. Anti-CD19 chimeric antigen receptor (CAR)-T cell therapy has revolutionized the treatment landscape of R/R LBCL. Currently, there are three anti-CD19 CAR-T products (axicabtagene ciloleucel, tisagenlecleucel, and lisocabtagene maraleucel) approved by the U.S. Food and Drug Administration (FDA) for the treatment of R/R LBCL. However, patients with SCNSL were excluded in these trials due to concerns for central nervous system (CNS) toxicity [1,2]. However, retrospective data have shown that patients with R/R SCNSL treated with axicabtagene ciloleucel or tisagenlecleucel could achieve high response rates; however, the durability of the response still remains unclear [3]. The TRANSCEND trial, which led to lisocabtagene maraleucel approval, did not exclude patients with SCNSL; however, only 3% of the patients had SCNSL [4]. As such, literature on the durability of the response of R/R SCNSL to CAR-T cell therapy remains sparse.

Here, we share our single institution experience of patients with R/R SCNSL treated with anti-CD19 CAR-T cell therapy using tisagenlecleucel.

## Case presentation

Patient 1

**Figure 1:**
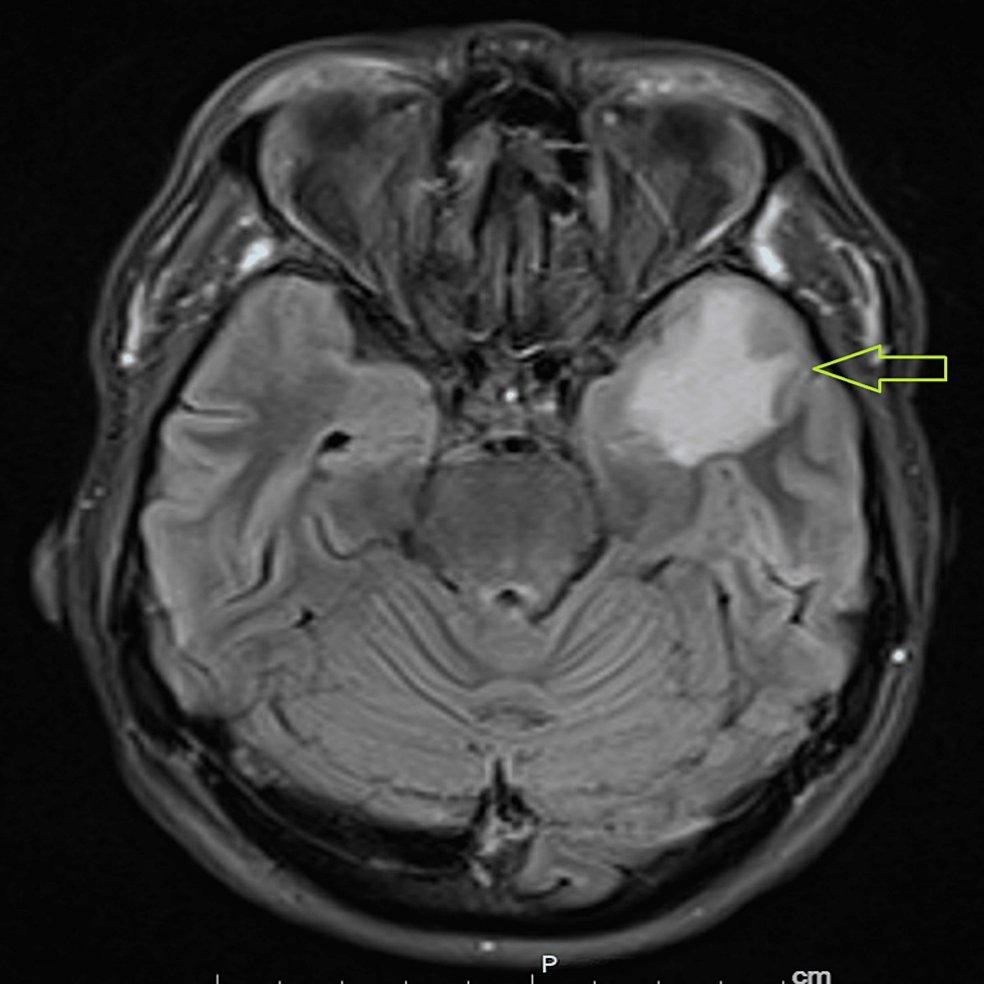
MRI of the brain demonstrating an intra-axial mass in the left temporal pole with associated parenchymal edema

A 51-year-old woman presented to the emergency department (ED) with the chief complaint of epistaxis and worsening headaches. The patient was found to have pancytopenia. Bone marrow biopsy revealed germinal center B-cell (GCB) diffuse large B cell lymphoma (DLBCL). Fluorescence in situ hybridization was positive for translocation of BCL2, BCL6, and MYC. Magnetic resonance imaging (MRI) of the brain revealed an intra-axial mass located within the left anterior temporal lobe with associated parenchymal edema (Figure 1).

The mass was biopsied, and pathology demonstrated diffuse large B cell lymphoma.

**Figure 2:**
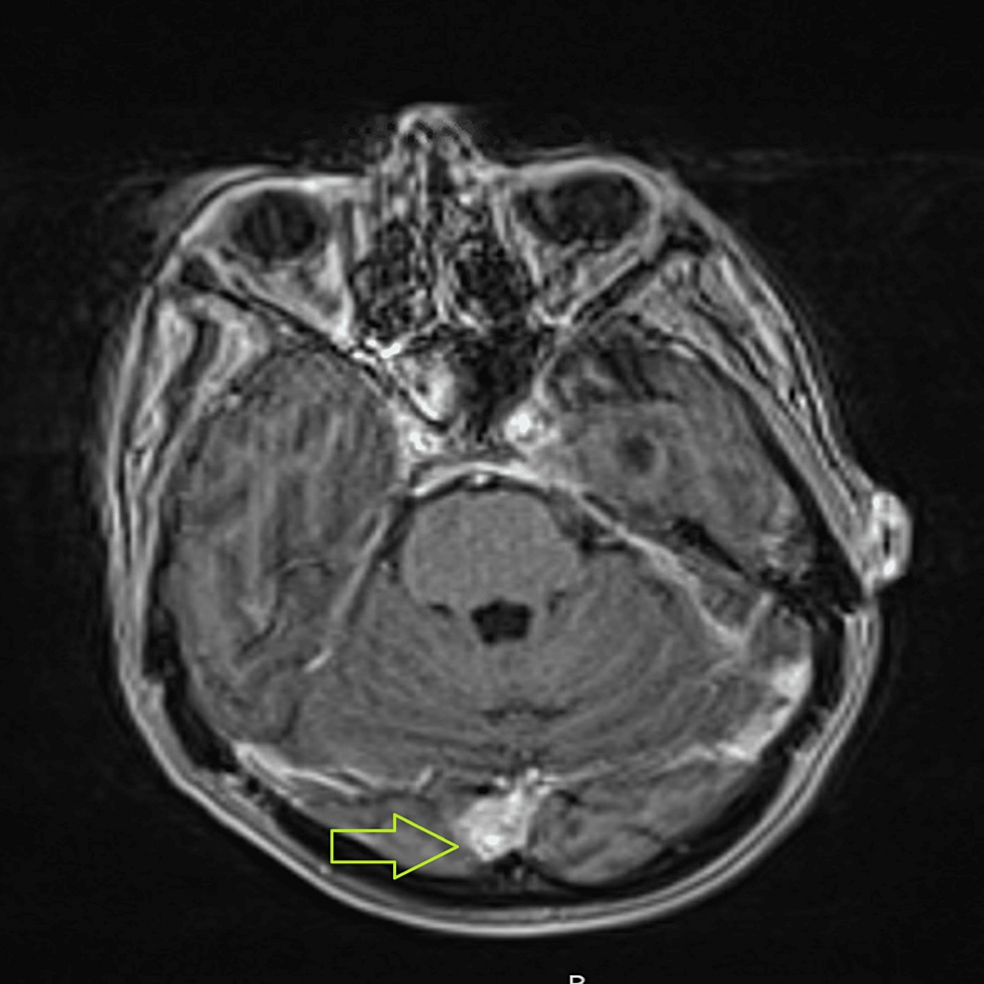
Three months post-CAR-T MRI of the brain revealed enhancement affecting left-sided Meckel's cave, cisternal trigeminal nerve, and left internal auditory canal, with increased prominence of enhancement into the left inner ear structures and along the left seventh nerve

The patient was treated with six cycles of dose-adjusted R- EPOCH (rituximab, etoposide, prednisone, vincristine, cyclophosphamide, and doxorubicin) and two cycles of HD-MTX. After treatment, the patient’s PET-CT (positron emission tomography-computed tomography) was negative for the disease but continued to have persistent CNS disease. Subsequently, the patient was treated with twice weekly alternating intra-Ommaya methotrexate and ara-C, which resulted in clearance of CNS disease. Three monthly follow-up MRI of the brain revealed enhancement affecting left-sided Meckel's cave, cisternal trigeminal nerve, and left internal auditory canal, with increased prominence of enhancement into the left inner ear structures and along the left seventh nerve, likely reflecting leptomeningeal spread of disease as evidenced (Figure 2).

With these findings, the patient was offered CAR-T therapy.

The patient subsequently received lymphodepletion chemotherapy with fludarabine 25 mg/m2 and cyclophosphamide 250 mg/m2 from day -3 to day -1, followed by CAR-T cell therapy with tisagenlecleucel. CAR-T course was complicated by immune effector cell-associated neurotoxicity (ICANS) grade 4, for which the patient was transferred to the medical intensive care unit (MICU) and treated with dexamethasone, methylprednisolone, and four doses of anakinra 100 mg subcutaneously. ICANS improved, and, finally, her neurotoxicity resolved.

An MRI of the brain two months post-CAR-T infusion revealed decreased but persistent enhancement of the trigeminal nerve. Findings were consistent with a partial response (PR). However, the patient had recurrent peripheral adenopathy, which was proven to be relapsed DLBCL on biopsy. The patient was then offered salvage therapy with pembrolizumab. However, the disease continued progressing, and she was subsequently transitioned to hospice. The patient died two months after receiving CAR-T therapy.

Patient 2

**Figure 3:**
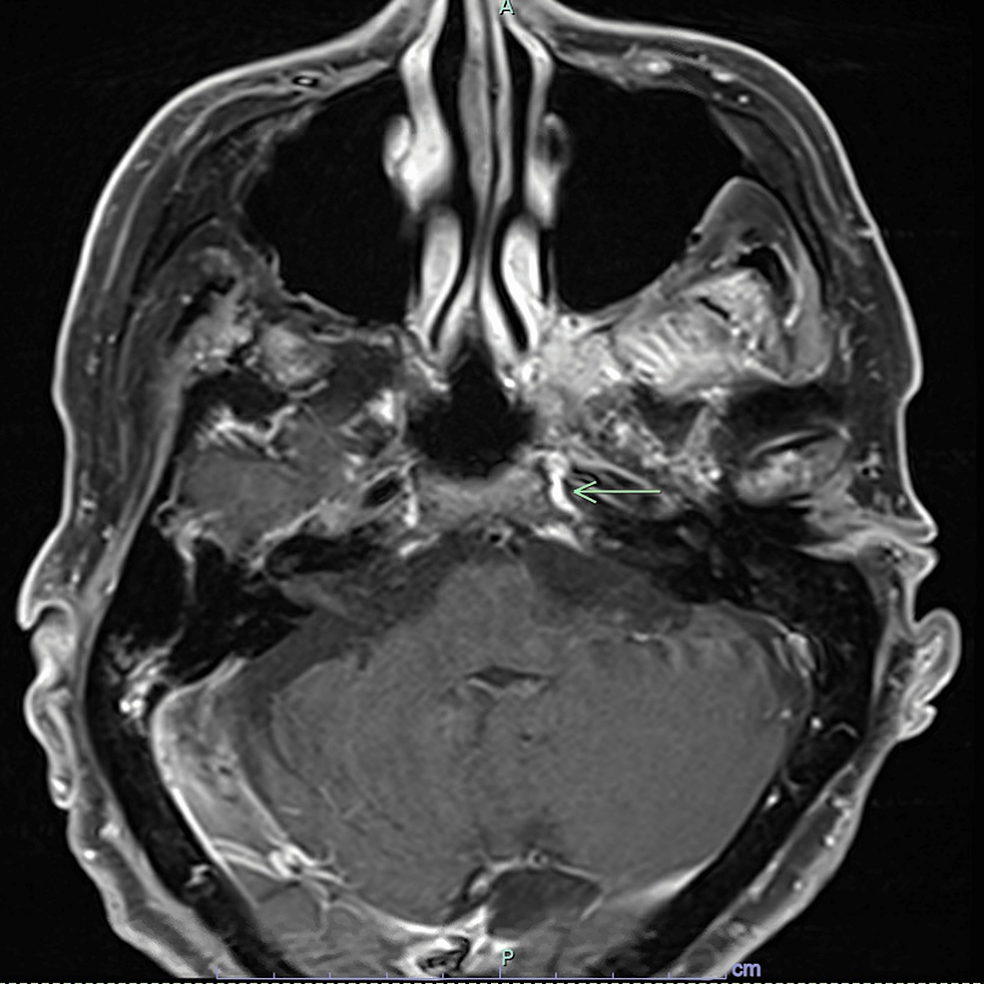
MRI of the brain revealed an enhancing lesion of the right intracranial prechiasmatic optic nerve

A 70-year-old man developed a non-healing oral ulcer, which was subsequently biopsied and revealed GCB-DLBCL, negative for translocations. The patient was started on R-CHOP (rituximab, cyclophosphamide, vincristine, and prednisone) chemotherapy, and after the second cycle, he developed acute vision loss. MRI of the brain revealed an enhancing lesions of the right intracranial prechiasmatic optic nerve (Figure 3), which was proven to be DLBCL on biopsy. The patient was then treated with WBRT along with single-agent rituximab. Follow-up imaging revealed persistent disease, and the patient was subsequently offered HD-MTX. The patient’s disease failed to respond, and treatment was changed to alternating intrathecal MTX and cytarabine. Follow-up imaging showed primary refractory disease in both the CNS and periphery. As such, the patient was subsequently offered CAR-T therapy with tisagenlecleucel.

Bone marrow biopsy and PET-CT imaging performed on day 30 revealed complete remission. However, PET-CT and brain MRI performed on day 100 revealed the progression of the disease. The patient was subsequently transitioned to tafasitamab and lenalidomide. However, no response to therapy was seen, and the patient died due to the progression of the disease.

Patient 3

A 65-year-old man with DLBCL treated with R-CHOP and HD-MTX presented with new-onset diplopia, with brain MRI revealing leptomeningeal enhancement on the left oculomotor nerve. He underwent a spinal tap, which was negative for lymphoma cells. He was started on high-dose cytarabine alternating with intrathecal methotrexate for three cycles.

A plan was made to initiate CAR-T treatment for his relapsed DLBCL with secondary CNS involvement. R-ICE (rituximab, ifosfamide, etoposide) was initiated for bridging purposes, and the patient completed three cycles of the regimen.

**Figure 4:**
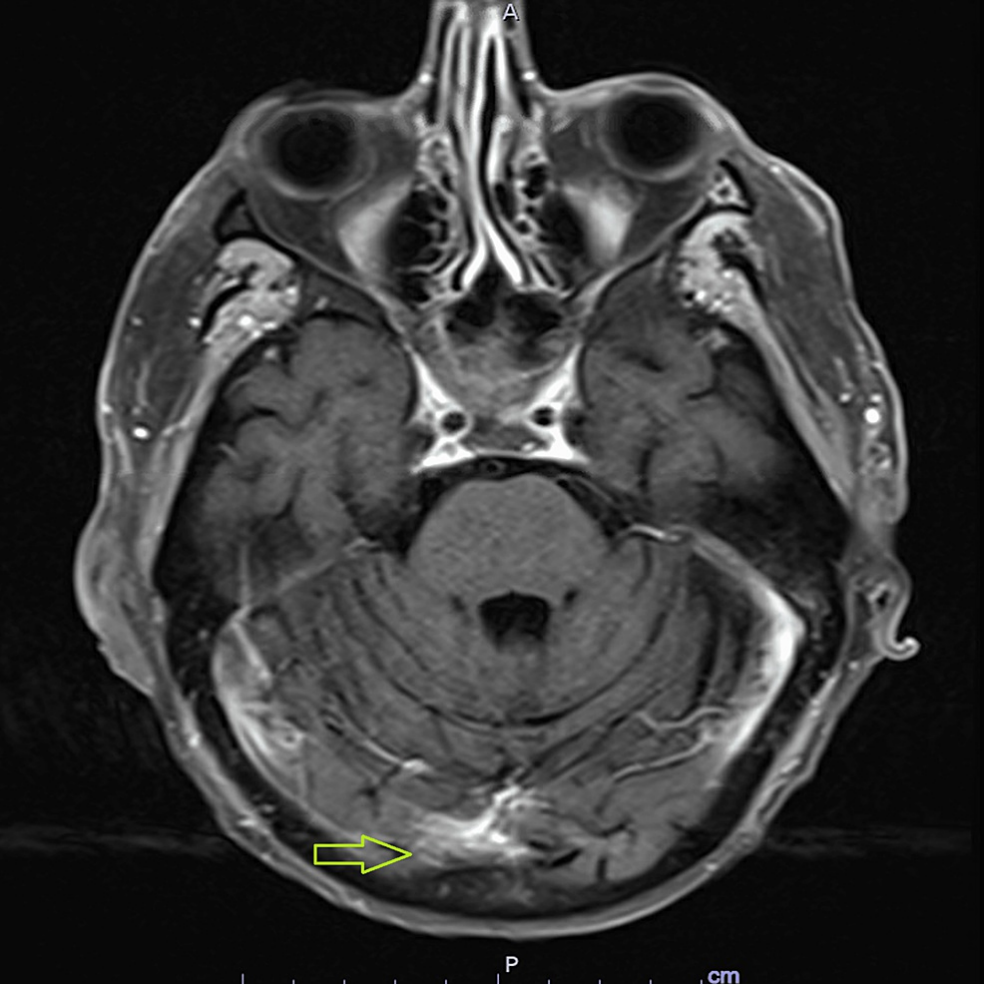
MRI of the brain demonstrating leptomeningeal enhancement of the superior and inferior cerebellar vermis

The patient received tisagenlecleucel. His post-CAR-T course was complicated by ICANS grade 4 and cytokine release syndrome (CRS) grade 1, necessitating treatment with four doses of tocilizumab and high-dose steroids. Additionally, he received a seven-day course of 100 mg of anakinra daily. His mental status continued to worsen, requiring MICU admission and intubation. His hospital admission was further complicated by pancytopenia, requiring multiple packed red blood cell and platelet transfusions. His bone marrow biopsy two weeks after CAR-T revealed complete remission. However, MRI brain findings showed persistent leptomeningeal enhancement (Figure 4).

Despite management with antimicrobials and steroids, the patient’s condition continued to deteriorate, leading to his ultimate demise 23 days after CAR-T cell therapy.

The three cases are described in Table 1.

**Table 1:** Demographics, outcomes, and management of adverse events of the three cases DLBCL, diffuse large B cell lymphoma; CNS, central nervous system; CAR-T, chimeric antigen T cell receptor therapy; CRS, cytokine release syndrome; ICANS, immune effector cell-associated neurotoxicity syndrome; WBRT, whole-body radiation therapy; GCB, germinal center B-cell; R-DA-EPOCH, rituximab, dose adjusted etoposide, prednisone, vincristine, cyclophosphamide, doxorubicin; HD-MTX, high-dose methotrexate; HD, high dose; R-CHOP, rituximab, cyclophosphamide, vincristine, and prednisone; R-GCVP, rituximab, gemcitabine, cyclophosphamide, vincristine, and prednisolone; ABC, activated B-cell; IT-MTX, intrathecal methotrexate; DA-ICE, dose-adjusted ifosfamide, carboplatin, etoposide

Age	DLBCL type	CNS disease location and status at the time of CAR-T	CNS symptoms	Systemic disease at the time of CAR-T	No. of previous lines of therapy	Systemic status before CAR-T	CAR-T product/dose	Bridging therapy	30-day response	CRS grade, treatment	ICANS grade, treatment	Current status	WBRT pre-CAR-T	Follow-up therapy
51	GCB	Parenchymal and leptomeningeal (active CNS disease)	Incidental	Absent	Two	Progressive disease	Tisagenlecleucel	No	Partial response	No	Grade 4, anakinra	Died two months after CAR-T	No	Pembrolizumab
					R-DA-EPOCH and HD-MTX		4.7x10^8^							
					HD cytarabine and IT-MTX									
					70		GCB								Leptomeningeal (active CNS disease)	Vision loss	Present	Four	Progressive disease	Tisagenlecleucel	No	Complete remission	No	No	Progressive disease	Yes	Tafasitamab and Lenalidomide
R-CHOP HD-MTX	5.4x10^8^																										
HD cytarabine and IT-MTX																											
R-GCVP																											
65	ABC	Leptomeningeal (active CNS disease)	Diplopia	Yes		Three		Progressive disease	Tisagenlecleucel	DA-ICE	NA	Grade 1, tocilizumab, dexamethasone taper	Grade 4, dexamethasone taper, anakinra	Died 23 days after CAR-T				No		None							
						R-CHOP and HD-MTX			5.1x10^8^																		
					High-dose cytarabine with IT-MTX															
					DA-ICE															

## Discussion

R/R DLBCL carries a dismal prognosis, with fewer than 50% of the patients displaying some response to subsequent treatments after second-line salvage treatment [5]. The prognosis worsens in patients with comorbidities, CNS involvement, and age >65 years. Due to a strict inclusion criterion, patients with CNS involvement in DLBCL were excluded from the large-scale CAR-T JULIET and ZUMA-1 clinical trials [6]. Patients with SCNSL were excluded from trials citing the risk of increased neurotoxicity in these patients. As such, data remain limited regarding the safety, efficacy, and duration of response of SCNSL to CAR-T therapy.

In our series, only one patient achieved a complete response (CR) 30 days post-CAR-T, but the 100-day imaging revealed the progression of the disease. The other two patients had a limited duration of response in CNS disease after CAR-T therapy, with one patient passing away due to CNS toxicity. The number of patients limits our series; however, it suggests that though CAR-T therapy may result in disease response in R/R SCNSL, the duration of response may be limited.

A study of five SCNSL patients treated with anti-CD19 CAR-T detected the presence of CAR-T cells in the cerebrospinal fluid (CSF), demonstrating the migration of these cells from the periphery to the CNS necessary for anti-lymphoma activity in the CNS [7]. On day 28, three of the five patients achieved CR, and the other two had stable disease. Of the three patients with CR, one continues to have a durable response 520 days post-CAR-T. Ahmed et al. reported their experience with seven patients with SCNSL treated with CAR-T therapy, with a CR of 85%, a median progression-free survival (PFS) of 83 days, and median overall survival of 129 days [3]. The other large-scale study by Bennani et al. reported outcomes in 17 patients who received axicabtagene ciloleucel for SCNSL using data from the U.S. Lymphoma CAR-T Consortium with six months of reported PFS of 36 days [8]. Frigault et al. reported outcomes of 8 patients treated with tisagenlecleucel for SCNSL, showing CR of 25% and PR of 25% [9]. The outcomes of the above studies are given in Table 2. 

**Table 2:** Depiction of the response of SCNSL to CAR-T cell therapies in different studies. SCNSL, secondary central nervous system lymphoma; CR, completer response; PR, partial response; PD, progression of disease; CRS, cytokine release syndrome; ICANS, immune effector cell associated neurotoxicity syndrome; ASCT, autologous stem cell transplant; WBRT, whole body radiation therapy; allo-SCT, allogenic stem cell transplant

Study	Active no. of SCNSL patients at infusion	Patients with CR	Patients with PR	Patients with PD	CRS events (grade ≥ 3 events)	ICANS events (grade ≥ 3 events)
Bennani et al. [8]	5	2	1	2	4 (1)	5 (3)
Abramson et al. [4]	6	3	-	-	No severe CRS	2 (2)
Ahmed et al. [3]	7	4 (1 had ASCT, 3 had WBRT)	-	3 (1 had ASCT)	4 (1)	3 (1)
Frigault et al. [9]	8	2 (1 had allo-SCT)	2	4	7 (0)	1 (0)
Our series	3	-	-	3	1 (0)	2 (2)

## Conclusions

All the studies mentioned in this case series suggest that CAR-T-cell therapy can help achieve responses in patients with SCNSL without increased toxicity. Compared to these studies, the patients in our series did not have appropriate responses and eventually relapsed, resulting in death. Our case series highlights the challenge of the durability of responses and the possibility of considering post-CAR-T chemotherapy to potentiate or maintain response in CNS lymphoma patients. Future studies are needed to improve outcomes in this group of patients.
